# Medicine

**Published:** 1894-06

**Authors:** R. Shingleton Smith


### Progress of the Medical Sciences.

### MEDICINE.

Much progress has been made since the proofs of the existence of a phthisical contagion were reviewed in these pages in the year 1883, soon after the discovery by Koch of the bacillus which is especially associated with his name. My own summary of the experimental evidence on which the inseparable connection between the bacillus and tubercle was founded, and the clinical evidence which then existed, was followed by a powerful accumulation of clinical evidence collected by Dr. Markham Skerritt, who urged that every consideration should be given to the weighty evidence in opposition to the theory of contagion.[1] It is no longer necessary to give proofs of the existence of the contagion itself, or of the need for antiseptics in the treatment of the disease. The evidence on which Dr. William Budd[2] announced his belief that phthisis would be found to be contagious, and which led him to the conviction that the deposits of tuberculous matter are of the nature of an eruption, and

[1] *Bristol M.-Chir. J.*, vol. i., 1883, pp. 1—70. [2] *Lancet*, 1867, vol. ii., p. 451.

bear the same relation to the disease as the yellow matter of typhoid fever bears to the fever itself, led him also to the logical conclusion that "by the destruction of tubercular matter in its issue from the body, by means of proper chemicals and otherwise, succeeded by good sanitary conditions, there is reason to hope that we may eventually, and possibly at no very distant date, rid ourselves entirely of this fatal scourge." We are even yet far off from this long-looked-for consummation, and, in spite of constantly repeated experimental demonstrations, and of an overwhelming mass of accumulated clinical evidence in animals and man, the profession as a whole has not yet adopted the logical methods of treatment which necessarily follow from an ungrudging and hearty acceptance of the truths which have been so amply proved.

The reason for this slowness in the acceptance of verified facts arises mainly from the limitations in the action of the specific virus. It has been emphasised by Daremberg[1] that few of those who may be exposed to it are found to be favourable soil; if it were otherwise, "if the contagion of phthisis was necessarily fatal, if no organism could escape it, then humanity itself would have been long since destroyed."

And yet the tubercle bacillus has become the one fixed fact in the pathology of phthisis, the acknowledged centre about which all the phenomena revolve; the study of the etiology of tuberculosis has become that of the life history of the bacillus, the symptomatology becomes the effects of the bacillus in the body, the definite diagnosis becomes the recognition of the bacillus in the discharges or the tissues, and the therapeusis becomes resolved into the use of agents which destroy or prevent its growth.[2]

A paper by Dr. T. Mitchell Prudden, on the element of contagion in tuberculosis,[3] points out that the modern view of an infectious disease is one which is caused by the invasion and reproduction within the body of pathogenic micro-organisms, and that "*infection* is the condition produced by the entrance and multiplication of pathogenic micro-organisms within the body. . . . The *contagium* in any infectious disease is . . . the particular pathogenic micro-organism itself . . . An infectious disease is contagious when its contagium . . . under the ordinary conditions of life, can be freed from the body of a diseased person, and, by whatever means, conveyed to the body of another in a condition capable of lighting up the disease anew. The old indefinite distinction between infection and contagion, by which one strove to express, among other things, a fundamental difference between the conveyance of disease by personal contact and by aërial transmission, has become impracticable and valueless now, because we know to-day that

[1] *Traitement de la Phtisie pulmonaire*, vol. i., 1892, p. 54.
[2] Overholser, *Med. Rec.*, vol. xliv., 1893, p. 291.
[3] *Tr. N. York Acad. M.*, vol. ix., 1893, p. 19.

the differences in the mode of communicability of infectious disease are largely dependent upon the physical qualitie of the contagia, upon the places and ways in which these are freed from the body, and upon the places and ways in which they enter the bodies of new victims."

Dr. E. G. Janeway,[1] in a paper on "The Necessity and Feasibility of Efforts to Prevent the Spread of Pulmonary Tuberculosis," further defines our position on this question in the following paragraph: "We cannot believe that the bacillus of tubercle will cause the development of tuberculosis in the human body, without becoming converted to the doctrine of contagion, at least in the sense that such bacilli eliminated from one person are liable to be received by another and, meeting with favourable conditions, establish the disease in the recipient. This granted, all is granted: for it practically admits contagion."

Dr. John S. Billings [2] points out that of the various infected localities "a specially infected and dangerous locality is a criminal court-room. Into this are brought every day persons affected with this disease, and depositing infectious sputa on the floors, where they soon dry and are ground into dust which pervades the air. These places, being never effectually cleaned from the bacteriological point of view, become centres of infection, causing disease not only in prisoners, but in spectators and lawyers."

A further paper by Dr. J. West Roosevelt, on "Practicable and Impracticable Plans for Diminishing the Spread of Phthisis Pulmonalis,"[3] sums up the two questions: "What ought to be done?" and the more practical one, "What can be done?" He points out the folly of advocating "measures which are so radical, or expensive, or complicated and troublesome that the majority of people will never attempt to carry them out," and the futility of attempting to destroy every bacillus in the world. Hence the necessity for full consideration of all the questions relating to susceptibility and the soil. "The least impartial must confess that tuberculosis is certainly not contagious for all men alike, but broadly speaking only to those who, as we express it, have a predisposition towards the disease."[4] "We cannot hope to destroy the ectanthropic sources of infection since they are too widely distributed, nor can we destroy the affected individuals, since such destruction must always be incomplete, unavailing, even if free from other objections. . . . We must rely chiefly on the removal of the special predisposition, counteract the causes responsible for such predisposition, and, at the same time, destroy as far as possible all ectanthropic sources of infection."[5]

Dr. Chaplin [6] has formulated the conclusion that the "prevention of phthisis can only be effected by the strictest

1 *Tr. N. York Acad. M.*, vol. ix, 1893, p. 26. 2 *Ibid*, p. 36. 3 *Ibid*, p. 47.

4 Kanthack, *Med. Mag.*, Oct., 1893. 5 *Ibid*. 6 *Med. Mag.*, May, 1893.

measures, such as notification, segregation, and interdiction of marriage;" whilst, on the other hand, Dr. Goodhart[1] comes to an entirely different conclusion, and negatives these more rigorous measures.

Dr. Mitchell Prudden[2] does not see why the spread of tuberculosis should not continue if the present insanitary habits continue. "If the vile and increasing practice of well-nigh indiscriminate spitting goes on unchecked in nearly all assembling places and public conveyances; if the misguided women who trail their skirts through the unspeakable and infectious filth of the streets are to be admitted uncleansed into houses and churches and theatres; if theatres and court rooms and school houses and cars are to remain the filthy lurking places of contagia which their ill ventilation and their mostly ignorant and careless so-called cleaning necessarily entail; if in sleeping cars and hotel bedrooms the well are to follow consumptives in their occupancy without warning or even the poor show of official disinfection; if in ill-ventilated and ill-cared-for dwellings the well must breathe again and again the dust-borne seeds of tuberculosis; if no persistent warning is to be given to the ignorant of the dangers which lurk in uncleanliness—then our task will be most complex as well as difficult in limiting the contagiousness of tuberculosis." He concludes his paper as follows: "To make our way between the rigors of necessary legislation on the one hand and the demands of the humanities on the other is a task requiring tact as well as wisdom, and large knowledge withal of the daily ways of the world as it goes on outside of laboratories. But, wisely choosing thus the way with caution, let us not forget that death meanwhile holds carnival."

I have elsewhere[3] summed up the methods of dealing with tubercular phthisis from the point of view of antiseptics and contagion. We must urge "a consistent and methodical warfare against infectious dust."[4]

Dr. C. G. Currier[5] formulates the knowledge of to-day in this regard as follows:—

Keep clean. Avoid unclean places and unclean and diseased people.

Do not spit on the floor or on the ground, and do not allow others to do so.

Expectorated matter loses its harmfulness when burned.

Clothing and other articles used by "consumptives" can be sterilized by exposure to the heat of boiling water for at least fifteen minutes.

It is safest to use milk, water, and other foods, only after they have been well cooked.

Abundant fresh air is a valuable purifier.

1 *Med. Mag.*, June, 1893. 2 *Loc. cit.* 3 *Med. Ann.*, 1894 p. 462—73
4 *Brit. M. J.*, 1893, vol. ii., p. 633.
5 *Tr. N. York Acad. M.*, vol. ix., 1893, p. 44.

The following code of precautions against consumption is now in use at the Bristol General Hospital:—

When the matter which is coughed up in some kinds of chest complaints dries on the ground, the dust carries back the infection to the patient himself and to other people, although the breath of consumptive persons is not directly infectious.

Therefore—

(1) Patients must use spitting cups, in which liquid disinfectants should be kept. The contents should be emptied on the fire and the cups scalded daily in boiling water.

(2) They must never spit on the ground or out of doors. In place of handkerchiefs they should use pieces of rag which can be burnt or thoroughly boiled.

(3) No linen or handkerchief on which anything from the patient has fallen should be allowed to dry, but should be placed in carbolic lotion and thoroughly boiled.

(4) The phlegm must not be swallowed. The contents of a bed-pan should have a disinfectant poured on them at once. The cup, spoon and fork of a patient should be used by him alone, and be put into boiling water after each meal.

(5) Cows' milk should always be boiled before it is used by anyone. Consumptive mothers should not suckle their children.

(6) The bedroom of a consumptive patient must have plenty of fresh air day and night, and the chimney must be kept unstopped. The walls and boards of his room must be often wiped with a damp cloth to avoid raising any dust.

(7) Great cleanliness and plenty of sunlight and fresh air in the rooms are most important.

So much is the diagnosis of the early stages of enteric fever beset with fallacies that any trustworthy aid will be gladly welcomed, and hence the "diazo reaction" as a urinary test has received much attention. Ehrlich has ascribed to it a distinct diagnostic value; but on the other hand many observers believe that little reliance can be placed upon it. The latest addition to the extensive literature of the subject is from Dr. John Hewetson,[1] who, from an analysis of the results given in 196 cases, concludes that as a diagnostic aid "it fails us when most needed, and although its absence during the entire disease would seem, according to most observers, to be of very rare occurrence, it must not be forgotten that the substances in the urine which give rise to it may have been present and have disappeared before the patient has come under observation."

[1] *Johns Hopkins Hosp. Rep.*, vol. iv., 1894, p. 113.

"Cardiac Asthenia, or Heart-exhaustion," is the title of a paper by Dr. J. M. Da Costa.[1] He differentiates between the weak heart, as it is commonly known, which comes from organic causes, and with which everyone is familiar, and a condition in which for a long period there is habitual feeble action of the heart as the essential and only appreciable disorder. The distinction between these two conditions must be far from easy, but the author states that the diagnosis is not, as a rule, difficult. "The evident nature of the causes that have given rise to the heart-wreck, its generally sudden onset, the unembarrassed breathing, the feebleness of the pulse and of the cardiac impulse, are full of significance. The physical signs as well as the state of the respiration and the clinical history separate the weak asthenic heart from the weak heart of organic type, such as the typical ones of this group — fatty degeneration and cardiac dilatation."

Dr. A. B. Macallum has given us a lengthy research on the absorption of iron in the animal body,[2] and his results give substantial evidences in favour of the view that *inorganic* iron is the specific for chlorotic anæmia.[3] Clinical evidence has long favoured this view, and now we have the experimental evidence, showing that in guinea pigs there is absorption in the intestinal mucosa, which, after treatment with alcohol and when tested with ammonium sulphide, acquires a more or less dark colour, due to the formation of sulphide of iron, which, under the microscope, is seen to be limited to the sub-epithelial portions of the tips of the villi; on closer examination the iron is found to be deposited in leucocytes which, in their disposition, form together a cap as it were for the extreme end of the lacteal vessel. When the dose is small, absorption occurs only in that part of the intestine adjacent to the pylorus and measuring only a few inches in length; yet when the quantity given at one time is large, the absorptive area may embrace the whole of the small intestine. Here we have a clear explanation of the greater utility of the large doses.

The comparatively rare disease, known as desquamative enteritis or mucous colitis, has been described anew by Dr. E. M. Light,[4] who is not able to throw any fresh light on the cause of the affection, which, he says, is unknown. The results of treatment appear to have been satisfactory, as he states that "most cases can be cured by careful treatment." This is not my experience. In a period of twenty-five years I remember only four or five cases, and in these the results have been almost entirely unsatisfactory, inasmuch as no kind of treat-

[1] *Am. J. M. Sc.*, vol. cvii., 1894, p. 361. [2] *J. Physiol.*, vol. xvi, 1894, p. 268.
[3] See *Bristol M.-Chir. J.*, vol. xi., 1893, p. 104.
[4] *Practitioner*, 1893, vol. l., p. 173.

ment has had the least effect in controlling the malady. Further investigations, possibly from the bacteriological point of view, are much needed, and may afford us a more useful kind of treatment than as yet exists: I have on one case exhausted the whole list of intestinal antiseptics, but the disease continues its course uninfluenced by any of them.

R. SHINGLETON SMITH.

